# Supplementary figures and images for: Neuronal glycolytic reprogramming drives lethality via accelerated aging in a Drosophila model of tauopathy
Source: bioRxiv. 2025 Oct 27:2025.10.27.684860. Preprint. [Version 1] doi: 10.1101/2025.10.27.684860 (PMC12636451; doi:10.1101/2025.10.27.684860)

# Figure S1

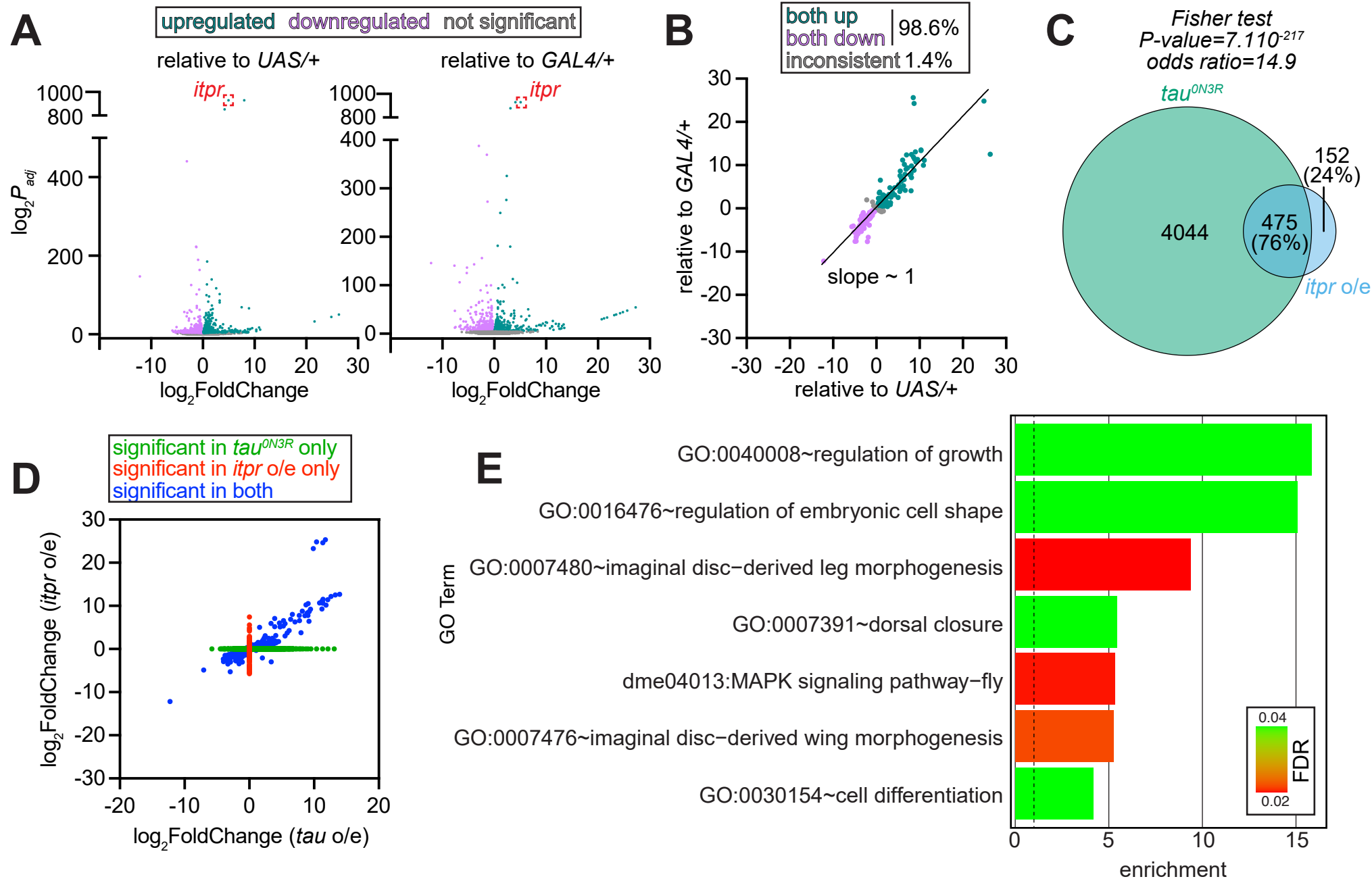

Supplement: Supplement 1 — (A) Volcano plots showing DEGs in larval brains overexpressing itpr (tubulin>itpr) relative to UAS-itpr/+ (left) and tubulin-GAL4/+ (right) controls. Upregulated genes (teal), downregulated genes (magenta), and non-significant genes (gray, adjusted P-value ≥ 0.05) are indicated. Also shown is the itpr gene (red), which is significantly upregulated. (B) Scatter plot comparing log2 fold changes of DEGs identified relative to each control line. High concordance (slope ~ 1) and 98.6% directional consistency demonstrate robust transcriptional changes induced by itpr overexpression. (C) Venn diagram showing overlap between tau0N3R DEGs (green, 4,519 genes) and itpr overexpression DEGs (blue, 627 genes). 475 genes (76% of itpr DEGs) are shared, representing a highly significant enrichment. (D) Scatter plot directly comparing log2 fold changes between DEGs in tau0N3R and itpr overexpression (o/e) for all genes. (E) Gene Ontology enrichment analysis of the 475 shared downregulated genes. Bar length represents fold enrichment, and color intensity indicates FDR. [file media-1.pdf]

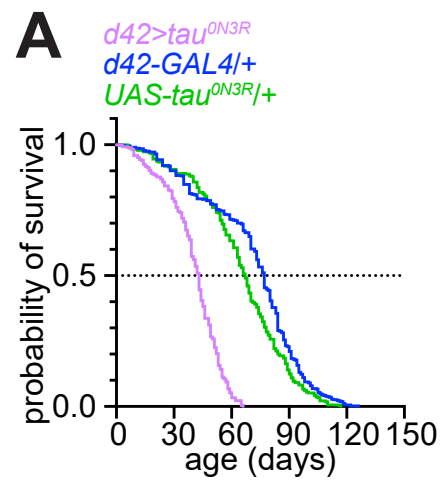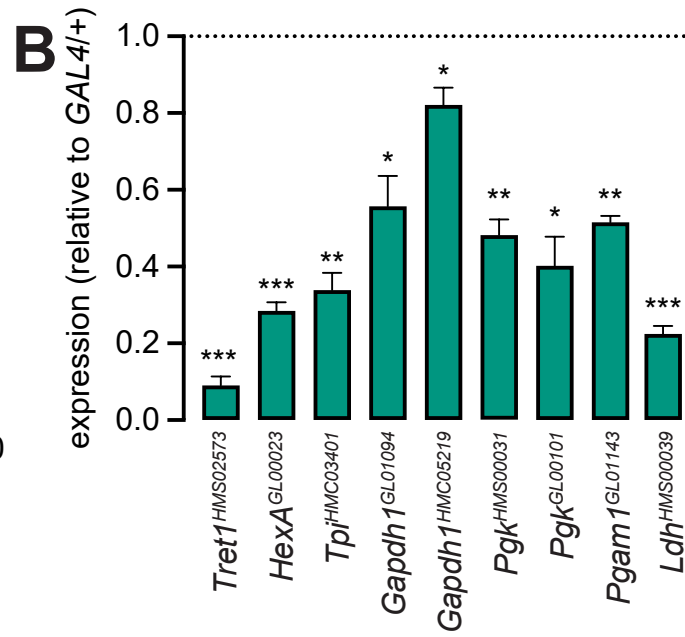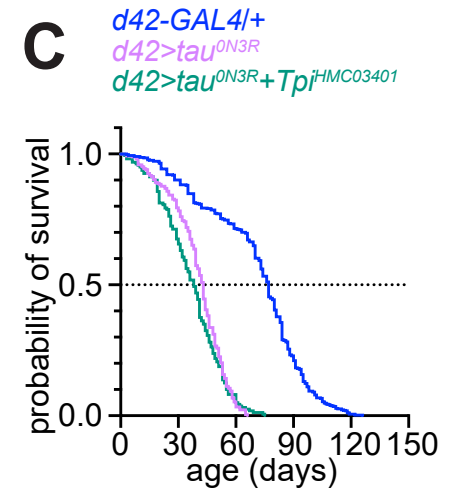

Supplement: Supplement 2 — (A and C) Kaplan-Meier survival curves in the indicated genotypes. Dotted line indicates 50% survival. See Supplemental Table 8A for sample sizes. Quantitative RT-PCR validation of RNAi-mediated transcript. All values are normalized to GAL4/+ controls and represent mean ± SEM, n = 3–6 biological replicates. *P < 0.05, **P < 0.01, ***P < 0.001, ****P < 0.0001, one-sample t-tests against control mean (1.0). [file media-2.pdf]

Figure 3

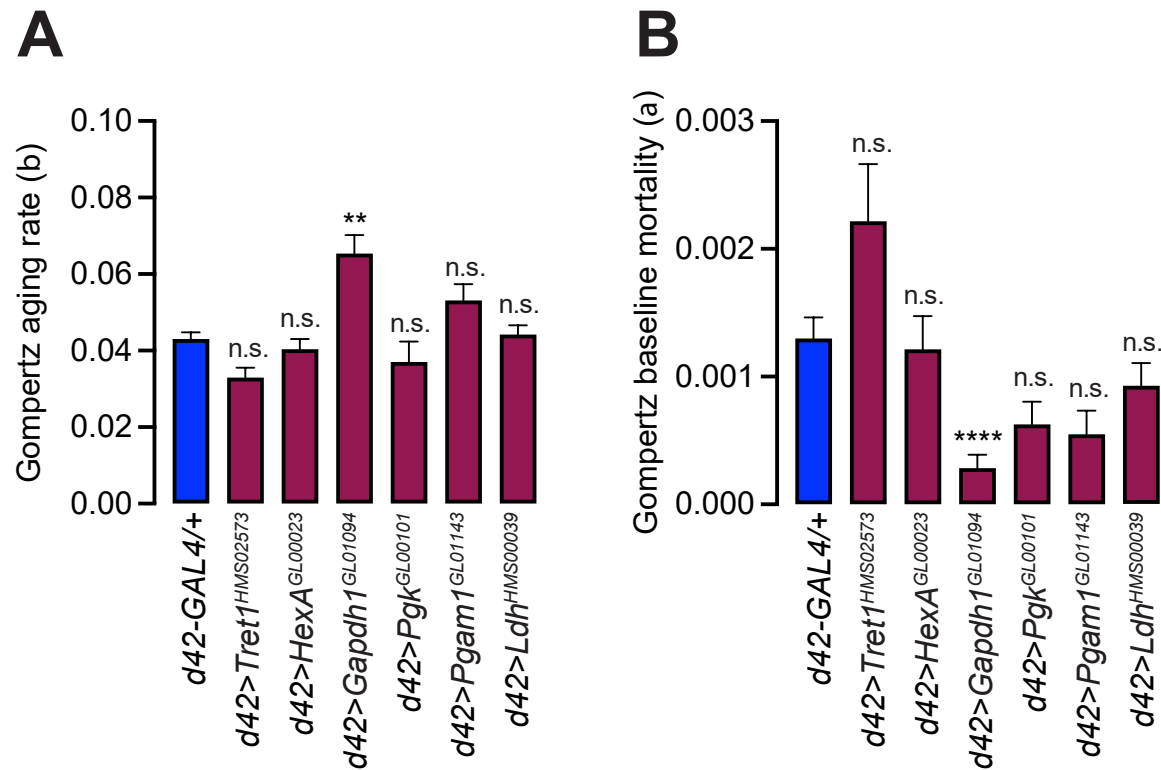

Supplement: Supplement 3 — (A-B) Gompertz aging rate parameter (A) and baseline mortalities (B) in the indicated genotypes. Values represent mean ± standard error from Gompertz model fits on lifespan data. All statistical comparisons were made with d42>tau0N3R flies. ****P < 0.0001, **P < 0.01, n.s., not significant, Wald z-test on pairwise parameter differences with Holm post hoc correction for multiple comparisons. [file media-3.pdf]
